# Adiponectin and Interleukin-33: Possible Early Markers of Metabolic Syndrome

**DOI:** 10.3390/jcm12010132

**Published:** 2022-12-24

**Authors:** Jelena Nesic, Biljana Ljujic, Vesna Rosic, Aleksandar Djukic, Milenko Rosic, Ivica Petrovic, Nenad Zornic, Ivan P Jovanovic, Sara Petrovic, Svetlana Djukic

**Affiliations:** 1 Department of Internal Medicine, Faculty of Medical Sciences, University of Kragujevac, 34000 Kragujevac, Serbia; 2 University Clinical Center Kragujevac, 34000 Kragujevac, Serbia; 3 Department of Human Genetics, Faculty of Medical Sciences, University of Kragujevac, 34000 Kragujevac, Serbia; 4 Department of Histology and Embryology, Faculty of Medical Sciences, University of Kragujevac, 34000 Kragujevac, Serbia; 5 Department of Pathophysiology, Faculty of Medical Sciences, University of Kragujevac, 34000 Kragujevac, Serbia; 6 Faculty of Medicine, University of Novi Sad, 21000 Novi Sad, Serbia; 7 Institute of Cardiovascular Diseases Vojvodina, Clinic of Cardiovascular Surgery, 21208 Sremska Kamenica, Serbia; 8 Department of Surgery, Faculty of Medical Sciences, University of Kragujevac, 34000 Kragujevac, Serbia; 9 Center for Molecular Medicine and Stem Cell Research, Faculty of Medical Sciences, University of Kragujevac, 34000 Kragujevac, Serbia; 10 Department of Infectious Diseases, Faculty of Medical Sciences, University of Kragujevac, 34000 Kragujevac, Serbia

**Keywords:** metabolic syndrome, obesity, insulin resistance, adiponectin, interleukin-33

## Abstract

Adiponectin is one of the most important molecules in the body’s compensatory response to the development of insulin resistance. By trying to maintain insulin sensitivity, increase insulin secretion and prevent inflammation, adiponectin tries to maintain glucose homeostasis. Interleukin-33, which belongs to the group of alarmins, also promotes insulin secretion. Interleukin-33 might be either pro-inflammatory or anti-inflammatory depending on the disease and the model. However, interleukin-33 has shown various protective effects in CVD, obesity and diabetes. The aim of our study was to investigate the association between adiponectin and interleukin-33 in patients with metabolic syndrome. As expected, all patients with metabolic syndrome had worse parameters that represent the hallmark of metabolic syndrome compared to the control group. In the subgroup of patients with low adiponectin, we observed less pronounced characteristics of metabolic syndrome simultaneously with significantly higher values of interleukin-33 compared to the subgroup of patients with high adiponectin. Our findings suggested that adiponectin might be an early marker of metabolic syndrome that emerges before anthropomorphic, biochemical and clinical parameters. We also suggest that both interleukin-33 and adiponectin may be used to predict the inflammatory status in the early stage of metabolic syndrome.

## 1. Introduction

The primary event in metabolic syndrome (MS) development is a chronic positive calorie intake with an increase in adipose tissue mass [1]. Central obesity is followed by the growth of insulin resistance (IR), one of the major mechanisms in the development of prediabetes and type 2 diabetes. (T2D) [2]. Simultaneously, a chronic low-grade inflammation of adipose tissue is accompanied by an increased level of pro-inflammatory cytokines [3]. The visceral adipose tissue (VAT) produces major amounts of adipocytokines that play a significant role in regulation of glucose, the metabolism of lipids and lipoproteins, blood pressure, hemostasis, and angiogenesis [4].

Adiponectin is produced by adipocytes and has insulin sensitizing, anti-inflammatory, antioxidative and antiapoptopic properties. Adiponectin enhances insulin secretion by stimulating both the expression of the insulin gene and the exocytosis of insulin granules. Adiponectin also acts in the brain to increase energy expenditure and may thereby promote weight loss. Adiponectin is independently and negatively related to MS, IR, T2D, body weight, blood pressure and serum lipids [5,6,7,8]. Since it was identified in the 1990s, adiponectin has been considered mostly as an anti-inflammatory adipokine, being able to induce the production of interleukin (IL)-10 and reduce tumor necrosis factor (TNF) in macrophages [9]. However, adiponectin belongs to a family of proteins with pro-inflammatory functions, and shares structure homologies with both TNF and immune complement protein C1q [10]. Previously, results suggest that adiponectin is able to initiate pro-inflammatory responses in cells from non-inflamed subjects and supports the hypothesis that adiponectin is implicated in the early phases of rheumatoid arthritis pathogenesis [11].

Interleukin-33 (IL-33) is an alarmin cytokine from the IL-1 family, which plays a crucial role in the initiation of Th2 immune responses as in metabolism regulation [12]. It is produced by mesenchymal cells in pancreatic islets and promotes insulin secretion. Thus, IL-33 contributes to the regulation of islet β-cell function [13]. IL-33 might be either pro-inflammatory or anti-inflammatory depending on the disease and the model. However, IL-33 was shown to have various protective effects in CVD, obesity and diabetes. Reduced levels may increase the risk of developing insulin resistance [14,15]. The main role of interleukin-33 and adiponectin in obesity-related diseases was shown in Supplement Table S1.

On the other side, it seems that these results are questionable in obesity-associated inflammation [16]. Interestingly, there is no or little data about the mutual relationship between IL-33 and adiponectin in diabetic and metabolic patients. Only one study was reported (Tabak et al. (2017)), and there was no association between IL-33 and adiponectin in patients with MS [17]. The contradictory results between this study and others may be linked to the different stages of the MS in the observed population.

Due to the insufficiently elucidated role of adiponectin and especially IL-33 and their interrelationship, the aim of this study was to examine the potential relationship and their role as a marker in the early diagnosis of patients with MS. It is conceivable that IL-33 and adiponectin might contribute to the development of MS and may also represent novel MS components. Future clinical studies are needed to confirm and extend these data.

## 2. Materials and Methods

Investigation was conducted as cross-sectional study which enrolled 112 subjects, 67 patients with diagnosis of MS and 45 healthy controls. Including criteria were over 18 years of age, an informed consent form was signed (after sufficient time after detailed information on the study), and diagnosis of MS. Diagnosis was confirmed according to The International Diabetes Federation consensus definition [18]. [On the other side, the exclusion criteria were previously known diabetes mellitus, subjects with oral antidiabetics, obesity drugs, glucocorticosteroids, immunomodulatory drugs, antipsychotics and antidepressants, subjects who had an acute infection in the last 2 weeks, subjects with malignancies within the past 5 years, liver and kidney failure.

### 2.1. Anthropometric Measurements

The body height of all study participants was measured using by Martin type anthropometer with precision of 0.1 cm, while the body weight was determined by a medical scale. The body mass index (BMI) was calculated according to the following formula: body weight (kg)/height (m)^2^. The waist circumference (WC) was measured at the end of expirium in the middle of the distance between the lowest point of ribs and the highest point of the femoral ridge of pelvis by a centimeter tape. Blood pressure (BP) will be measured in all subjects during the clinical examination.

### 2.2. Laboratory Analyses

Venous blood samples were collected after 12 h of fasting to determine the clinical parameters of inflammation (CRP, fibrinogen), lipid profile (total cholesterol, HDL, LDL, triglycerides), and glycosylated hemoglobin A1C (HbA1c). An oral glucose tolerance test (OGTT) was performed after the fasting period of 12 h with 75 g of glucose. Glycemia and insulinemia were measured just before the glucose load and then at 30, 60 and 120 min.

### 2.3. Enzyme-Linked Immunosorbent Assay (ELISA)

After sampling, the plasma samples were stored at a temperature of -80 degrees until analysis. Adiponectin HMW (Elabscience, Houston, TX, USA catalogue number E-EL-H5621), and interleukin-33 (R&D Systems, Minneapolis, MN, USA catalogue number DY3625) levels were determined by the ELISA method following the manufacturer’s instructions.

### 2.4. Hybrid Indices for the Evaluation of Metabolic Status

Insulin sensitivity was determined by calculating the Matsuda index. The homeostatic model assessment for insulin resistance index (HOMA-IR Index) was determined to calculate the level of insulin resistance. All listed indices are free and available at the following web address: http://mmatsuda.diabetes-smc.jp/MIndex.html (accessed on 27 October 2020).

### 2.5. Power of the Study

We calculated the power of the study using an appropriate computer program (G Power software 3.2.7) and a two-way *t*-test for independent samples with a 1:1 distribution of respondents by group. With an effect size of 0.86 (calculated from a study by Kowalska et al. [19]) a study power of 90% and a probability of type 1 error (α) of 0.05 was calculated with a total sample size of 60 subjects and at least 30 subjects in each group. The study design was based on the study by de Luis at colleagues [20]. 

### 2.6. Statistical Analysis

Statistical analyses were conducted by using the standard statistical package. The distribution of variables was assessed by Shapiro-Wilk test. *T*-test or non-parametric Mann-Whitney-U test were used to analyze differences between the groups. The effect of other parameters on adiponectin concentration was determined using linear regression with a confidence interval (CI) of 95%. All results were considered significant if *p* was lower than 0.05. For the comparison of the three groups, we used the provided statistical tests like ANOVA, Kruskal-Wolis and Man-Whitney test. During the comparison of two or more groups, in order to reduce the error of the first α type, we used the Bonferroni correction with a significance of *p* < 0.025. To eliminate the influence of significantly different BMI on adiponectin and interleukin-33 levels between groups, a covariance analysis was performed.

## 3. Results

### 3.1. Demographic and Clinical Characteristics of Study Population

One hundred and twelve subject patients gave informed consent and were enrolled in the study. Our study included patients with MS and a control group. As expected, MS patients had significantly higher systolic and diastolic BP, WC, and BMI with a similar gender distribution and average age between groups (Table 1).

### 3.2. Laboratory Characteristics

As expected, patients with MS had significantly worse all laboratory parameters of liporegulation, glycoregulation and inflammation compared to the control group. A significant increase of insulin resistance with a decrease in insulin sensitivity were also found. Differences in the concentration of IL-33 between the study groups were not found but a significantly higher concentration of adiponectin was observed in the group of patients with MS (Table 2).

The difference in BMI between the two groups is statistically significant, which may affect the significance of the differences in certain parameters between the groups, especially interleukin-33 and adiponectin. Therefore, a covariance analysis was performed, which eliminates the influence of BMI (Table 2 (b)).

### 3.3. Demographic and Clinical Characteristics of Subgroups Divided by Adiponectin Median

In order to examine the relationship between adiponectin levels and other parameters, the group of patients with MS was divided into two subgroups according to the median adiponectin (80 ng/mL). The difference in gender prevalence and age between the observed groups was not observed. The values of systolic BP, BMI and WC were significantly higher in subgroup of patients with MS and low adiponectin compare to the control group. In observed parameters, there was no significant difference between subgroups with MS. (Table 3).

### 3.4. Laboratory Characteristics of Subgroups Divided by Adiponectin Median

All observed parameters of liporegulation except LDL, all observed parameters of glycoregulation except insulinemia in 30 min and HbA1c, concentrations of IL-33 and adiponectin were significantly higher in the subgroup of patients with MS and low adiponectin compared to the control group. All mentioned parameters in the subgroup with MS and low adiponectin were equally expressed in the subgroup of patients with MS and high adiponectin. At the same time, significantly higher insulin levels at 30 min and HOMA index were observed in the subgroup of patients with MS and high adiponectin. These results clearly indicate a more severe stage of MS with compensatory hyperinsulinemia in this subgroup. The more severe stage of MS was accompanied by a significant increase in adiponectin and a decrease in IL-33. (Table 4).

### 3.5. Linear Regression between IL-33 and Adiponectin

Linear regression showed that IL-33 is an independent predictor in the model β −0.458; *p* 0.024 (95% CI) in the subgroup with low adiponectin concentrations of MS patients. Adiponectin concentration decreased 0.458 ng/mL for each nanogram per milliliter of IL-33 increase.

## 4. Discussion

In the present study, we showed higher values of adiponectin in patients with MS compared to the control subject. Moreover, in the subgroup of patients with low adiponectin, we observed significantly less pronounced characteristics of MS simultaneously. Additionally, our data show that the patients with low adiponectin had significantly higher values of interleukin-33 compared to the subgroup of patients with high adiponectin.

In contrast to other adipokines, adiponectin was established as an anti-atherogenic, anti-inflammatory, and anti-diabetic adipokine [21]. The beginning of the 21st century brought significant interest in adiponectin functions. Adiponectin levels in MS can vary and depend on factors such as gender, hormonal status, and circadian rhythm of secretion [22]. Sheng et al. have shown that low levels of adiponectin are associated with the development of T2D by increase of IR, increased gluconeogenesis, and finally a decrease in glucose uptake in liver and skeletal muscles [23]. Kern et al. examined adiponectin levels in varying degrees of obesity and insulin resistance in MS. Their results showed a significant association of adiponectin in plasma with adiponectin mRNA levels and that obese individuals expressed significantly lower levels of adiponectin [24]. Lindberg et al. also showed that decline in adiponectin is associated with an increased risk of MS over time [25]. On the other side, Yamauchi et al. demonstrated that continuous systemic infusion of a recombinant adiponectin (in physiological dose) reversed insulin resistance of lipoatrophic mice [26]. Systematic review and meta-analysis by Li et al. clearly showed that elevated adiponectin values were associated with a lower risk of T2D across diverse populations [27]. These results were confirmed by a recent conducted study by Lindberg et al., who showed that elevated adiponectin was associated with reduced risk of CV events [28]. Knowing adiponectin function, high values in the group of patients with MS should be interpreted as a compensatory increase to prevent the development of severe disorders of glucose metabolism. Significantly elevated values of solely HMW sub fraction of adiponectin in patients with type 1 diabetes (T1D) can be interpreted as confirmation of this compensatory increase. Although the pathogenesis of T1D and MS is significantly different, in both cases there is no effect of insulin on peripheral tissues, which leads to a compensatory increase of adiponectin [29]. 

After dividing patients into subgroups according to the median adiponectin (80 ng/mL), our results showed that adiponectin levels were similar between the low adiponectin subgroup and control group. Moreover, fine differences analysis showed significantly higher insulinemia and higher degree of IR in the high adiponectin subgroup. At the same time, a significant decrease in IL-33 with an increase in adiponectin was observed in the same subgroup.

Interleukin-33 is a tissue-derived nuclear cytokine from the IL-1 family with important roles in the maintenance of tissue homeostasis achieved by activating mast cells, ILC2s and Treg cells [30]. In adipose tissue of lean subject, IL-33 induces adipose-resident ILC2s to produce the Th2 cytokines and support the accumulation of eosinophils and M_2_ macrophages. Alternatively activated macrophages’ products, such as IL-10, contribute to an increasing in adipocyte insulin sensitivity and act protectively against T2D. Contrary, in the obese subjects, the selective accumulation and activation of adipose-resident group 1 innate lymphoid cells by IFN-γ production promote obesity-associated insulin resistance and T2D [31]. In pancreatic islets in diabetic or obese subject, the islets are in the state of chronic low grade inflammation. Interleukin-33 produced by mesenchymal cells, activate islet-resident ILC2s, which stimulate the capacity of myeloid cells to produce retinoic acid. An increased production of retinoic acid enhances insulin secretion in islet β cells and protects against T2D. In other words, normal or increased concentrations of IL-33 prevent the onset of diabetes [31]. 

Our results shoved negative correlation between IL-33 and adiponectin levels in low adiponectin subgroup patients with MS. Compared to the control group, in the subgroup of patients with MS and low adiponectin, we observed slightly higher values of IL-33 followed by slightly lower adiponectin (but without sufficient statistical significance). In the high adiponectin subgroup, we had significantly lower IL-33, significantly higher adiponectin levels with a more pronounced characteristics of the MS.

It is known that adiponectin, in addition to the same effect as IL-33 to increase insulin secretion. Unlike IL-33, adiponectin has significant additional effects to maintain insulin sensitivity and prevent apoptosis of β cells by attempting to maintain glucose homeostasis during compensatory growth. Accordingly, we hypothesized that increased adiponectin secretion is a major compensatory mechanism that attempts to prevent the onset of glucose metabolism disorders after a Th1/Th2 ratio disorder caused by low-grade inflammation. The amplification of Th1 responses due to increasing low-grade inflammation leads to reduced production and effect of IL-33. This hypothesis is supported by our results that showed slightly higher concentrations of CRP and fibrinogen in the subgroup with high adiponectin compared to the other subgroup. The deterioration of metabolic milieu was accompanied by an increase in the Th1 response and a decrease in IL-33 concentration. This decrease was accompanied by a compensatory increase of adiponectin as an attempt to improve insulin sensitivity, preserve β cells and prevent the development of more severe disorders at the level of the glucose continuum and the occurrence of T2D.

The main limitation of our study is a relatively small number of patients per subgroup with MS. The main reason for this are the logistical difficulties for analyzes that are not part of routine practice.

## 5. Conclusions

Our findings suggested that IL-33 and adiponectin might be an early marker of MS. We also suggest that both IL-33 and adiponectin may be used to predict the occurrence of significant hyperinsulinemia in the initial part of the second phase of insulin secretion, as well as the inflammatory status in MS patients, instead of others markers, which are a well-known acute phase inflammatory markers.

## Figures and Tables

**Table 1:** Clinical characteristics of patients with MS (n = 67) and control group (n = 45).

Characteristics * Mean ± SD	MS Group	Control Group	*p* **
Gender M/F	29/38	19/26	0.586
Age (years)	41.69 ± 15.55	33.08 ± 15.14	0.342
Systolic BP (mmHg) (Ref. < 120)	133.66 ± 22.77	115.80 ± 13.90	**<0.0005**
Diastolic BP (mmHg) (Ref. < 80)	85.36 ± 12.80	78.48 ± 9.26	**<0.01**
WC (cm)	108.46 ± 11.91	83.15 ± 8.88	**<0.0005**
BMI (kg/m^2^) (Ref. < 24.9)	32.93 ± 5.58	23.16 ± 3.70	**<0.0005**

* M-Male, F-Female, BP-blood pressure, WC-waist circumference, BMI- body mass index. ** The bolded results indicate statistically significant differences.

**Table 2:** (a) Clinical characteristics of patients with MS (n = 67) and control group (n = 45). (b). Results of covariance analysis.

**(a)**				
**Characteristics** **Mean ± SD**	**MS Group**	**Control Group**	**Ref. Range**	*p* *
Cholesterol (mmol/L)	5.85 ± 1.28	4.94 ± 0.88	<5.2	**<0.0005**
Triglycerides (mmol/L)	2.32 ± 1.46	0.88 ± 0.28	<1.7	**<0.0005**
HDL (mmol/L)	1.19 ± 0.24	1.47 ± 0.29	>1.1	**<0.0005**
LDL (mmol/L)	3.58 ± 1.04	3.07 ± 0.72	<3.5	**0.004**
Glycemia 0 min (mmol/L)	5.32 ± 0.83	4.40 ± 0.37	<5.6	**<0.0005**
Glycemia 30 min (mmol/L)	9.14 ± 1.61	7.37 ± 1.41	/	**<0.001**
Glycemia 60 min (mmol/L)	9.43 ± 2.59	5.84 ± 1.59	/	**<0.0005**
Glycemia 120 min (mmol/L)	6.98 ± 2.44	4.75 ± 1.36	<7.8	**<0.0005**
Insulin 0 min (IU/mL)	10.71 ± 5.82	4.85 ± 2.03	<20	**<0.0005**
Insulin 30 min (IU/mL)	59.97 ± 43.81	39.83 ± 18.29	<40	**0.002**
Insulin 60 min (IU/mL)	77.71 ± 52.32	35.48 ± 14.85	<40	**<0.0005**
Insulin 120 min (IU/mL)	60.91 ± 50.13	21.28 ± 17.76	<40	**<0.0005**
HbA1c (%)	5.48 ± 0.44	5.20 ± 0.41	<5.7	**0.001**
HOMA IR index	2.62 ± 1.54	0.94 ± 0.40	<2.5	**<0.0005**
Matsuda index	4.87 ± 3.76	11.13 ± 6.14	>4.3	**<0.0005**
CRP (mg/L)	4.36 ± 3.74	1.35 ± 1.30	<5.0	**<0.0005**
Fibrinogen (g/L)	3.39 ± 0.64	2.72 ± 0.44	<4.5	**<0.0005**
Interleukin-33 (pg/mL)	34.44 ± 10.65	35.78 ± 7.57	/	0.548
Adiponectin (ng/mL)	75.61 ± 15.41	67.57 ± 18.31	/	**0.018**
**(b)**				
**Characteristics** **Mean ± SE**	**MS Group**	**Control Group**	*p* *	
Interleukin-33 (pg/mL)	34.13 ± 3.45	28.56 ± 3.37	0.293	
Adiponectin (ng/mL)	66.03 ± 3.18	76.72 ± 2.55	**0.024**	

* The bolded results indicate statistically significant differences.

**Table 3:** Demographic and clinical characteristics of subgroups of patients with MS divided by mean adiponectin (cut off 80.0 ng/mL).

Characteristics *	Control Group (n = 45)	Low Adiponectin Group (n = 34)	High Adiponectin Group (n = 33)	*p* **	*p* * 1 vs. 2	*p* * 2 vs. 3	
Gender M/F	19/26	15/18	14/20	**0.679**			
Age (years)	33.08 ± 15.14	39.96 ± 12.79	42.46 ± 12.15	**0.006**	0.026	0.330	
Systolic BP (mmHg) (Ref. <120)	115.80 ± 13.90	130.67 ± 18.77	136.66 ± 26.86	**<0.0005**	**<0.0005**	0.173	
Diastolic BP (mmHg) (Ref. <80)	78.48 ± 9.26	82.48 ± 10.43	89.20 ± 14.20	**0.001**	0.119	0.046	
WC (cm)	83.15 ± 8.88	107.90 ± 11.91	108.13 ± 11.92	**<0.0005**	**<0.0005**	0.914	
BMI (kg/m^2^) (Ref. <24.9)	23.16 ± 3.70	32.83 ± 5.87	32.60 ± 5.21	**<0.0005**	**<0.0005**	1.000	

* M-Male, F-Female, BP-blood pressure, WC-waist circumference, BMI- body mass index. ** The bolded results indicate statistically significant differences. Kruskal Wolis test **, Mann—Whitney U test *.

**Table 4:** Laboratory characteristics of subgroups of patients with MS divided by mean adiponectin (cut off 80.0 ng/mL).

Characteristics	Control Group (n = 45)	Low Adiponectin Group (n = 34)	High Adiponectin Group (n = 33)	Ref. Range	*p*	1 vs. 2 ***	2 vs. 3 ***
Cholesterol (mmol/L)	4.94 ± 0.88	5.70 ± 1.11	6.04 ± 1.45	<5..2	* 0.001	**0.002**	0.403
Triglycerides (mmol/L)	0.88 ± 0.28	2.30 ± 1.35	2.30 ± 1.63	<1.7	* <0.0005	**<0.0005**	0.751
HDL (mmol/L)	1.47 ± 0.29	1.24 ± 0.26	1.16 ± 0.22	>1.1	* <0.0005	**0.001**	0.242
LDL (mmol/L)	3.07 ± 0.72	3.39 ± 0.86	3.83 ± 1.19	<3.5	* 0.026	0.126	0.178
Glycemia 0 min (mmol/L)	4.40 ± 0.37	5.20 ± 0.93	5.37 ± 0.71	<5.6	* <0.0005	**<0.0005**	0.236
Glycemia 30 min (mmol/L)	7.37 ± 1.41	9.12 ± 1.67	9.14 ± 1.57	/	** <0.0005	**<0.0005**	0.999
Glycemia 60 min (mmol/L)	5.84 ± 1.59	9.21 ± 2.63	9.30 ± 2.50	/	** <0.0005	**<0.0005**	0.978
Glycemia 120 min (mmol/L)	4.75 ± 1.36	6.92 ± 2.21	6.74 ± 2.39	<7.8	* <0.0005	**<0.0005**	0.908
Insulin 0 min (IU/mL)	4.85 ± 2.03	9.04 ± 4.89	11.97 ± 6.28	20	* <0.0005	**<0.0005**	0.043
Insulin 30 min (IU/mL)	39.83 ± 18.29	47.19 ± 30.64	74.26 ± 52.00	/	* 0.003	0.619	**0.016**
Insulin 60 min (IU/mL)	35.48 ± 14.85	70.02 ± 45.72	85.66 ± 57.17	/	* <0.0005	**<0.0005**	0.323
Insulin 120 min (IU/mL)	21.28 ± 17.76	53.62 ± 46.12	66.81 ± 52.86	/	* <0.0005	**0.019**	0.056
HbA1c (%)	5.20 ± 0.41	5.46 ± 0.45	5.47 ± 0.41	<5.7	**0.008	0.28	0.959
HOMA IR index	0.94 ± 0.40	2.17 ± 1.32	2.93 ± 1.30	<2.5	* <0.0005	**<0.0005**	**0.015**
Matsuda index	11.13 ± 6.14	5.70 ± 4.04	4.17 ± 3.43	>4.3	* <0.0005	**<0.0005**	0.035
CRP (mg/L)	1.35 ± 1.30	4.60 ± 3.93	5.84 ± 3.39	<5.0	* <0.0005	**<0.0005**	0.501
Fibrinogen (g/L)	2.72 ± 0.44	3.25 ± 0.55	3.49 ± 0.71	<4.5	** <0.0005	**<0.0005**	0.241
Interleukin-33 (pg/mL)	35.78 ± 7.57	39.20 ± 10.34	26.22 ± 7.41	/	* 0.035	0.975	**0.021**
Adiponectin (ng/mL)	67.57 ± 18.31	62.76 ± 10.10	88.45 ± 6.34	/	* <0.0005	0.244	**<0.0005**

* The bolded results indicate statistically significant differences. Kruskal wolis test *, ANOVA **, Mann-Whitney U test ***.

## Data Availability

The data presented in this study are available on request from the corresponding author.

## Supplementary Materials

The following supporting information can be downloaded at: https://www.mdpi.com/article/10.3390/jcm12010132/s1, Table S1: The main role of interleukin-33 and adiponectin in obesity-related diseases [32,33,34,35,36,37].
