# Supplementary material for: Adiponectin and Interleukin-33: Possible Early Markers of Metabolic Syndrome
Source: J Clin Med. 2022 Dec 24;12(1):132. doi: 10.3390/jcm12010132 (PMC9821697; doi:10.3390/jcm12010132)
Supplement: Supplementary file 1 [file jcm-12-00132-s001.zip › jcm-2093953-supplementary.pdf]

Supplement Table S1. The main role of interleukin-33 and adiponectin in obesity-related diseases.

The main role of interleukin-33 and adiponectin	
Interleukin-33	Adiponectin
Role in host defense, tissue repair and maintaining immune homeostasis [32]	Local anti-inflammatory effects [34]
Regulation of lipid metabolism and adipose tissue homeostasis [14, 33]	Improves hepatic insulin sensitivity and hepatic lipid metabolism [35]
Promote insulin secretion [13]	Promotes insulin secretion and have a cytoprotective effects on the β–cell [36, 37]
